# A Spanish-Language Patient-Reported Outcome Measure for Trust in Pregnancy Care Clinician

**DOI:** 10.1001/jamanetworkopen.2024.60465

**Published:** 2025-02-18

**Authors:** Rose L. Molina, Maria Bazan, Michele R. Hacker, Anjali J. Kaimal, Margarita Alegría, Leonor Fernández, Jeanne-Marie Guise, Maria O. Edelen

**Affiliations:** 1 Department of Obstetrics and Gynecology, Beth Israel Deaconess Medical Center, Boston, Massachusetts; 2 Harvard Medical School, Boston, Massachusetts; 3 Department of Obstetrics and Gynecology, University of South Florida, Tampa; 4 Departments of Medicine and Psychiatry, Massachusetts General Hospital, Boston; 5 Department of Medicine, Beth Israel Deaconess Medical Center, Boston, Massachusetts; 6 Department of Surgery, Brigham and Women’s Hospital, Boston, Massachusetts

## Abstract

**Importance:**

Despite the importance of patient trust in health care, there are no patient-reported outcome measures (PROMs) for trust in their clinician that have been developed empirically in Spanish, which is the second most common language in the US.

**Objective:**

To develop and validate a Spanish-language PROM for trust in pregnancy care clinician.

**Design, Setting, and Participants:**

This cross-sectional study used a national online panel of patients who reported a Spanish language preference and had limited English proficiency and were currently pregnant or had given birth within the 12 months before the survey. Participants resided in the United States, and data were collected from January to May 2024.

**Exposures:**

Participants had clinical interactions during pregnancy and/or postpartum care. Data collected included demographics, *Confianza* (Trust) Scale candidate items, and 4 measures for concurrent validity evidence: the Trust in Physician Scale (TPS), the Mothers on Respect Index, the Edinburgh Postpartum Depression Scale (EPDS), and the Patient-Reported Outcomes Measurement Information System Global 10.

**Main Outcomes and Measures:**

The main outcomes were psychometric properties of the Confianza scale and its association with validated scales (validity coefficients). Item response theory (IRT) analyses were conducted to evaluate the psychometric properties of the candidate items, select the best item subset for the Confianza scale, examine its correlation with other measures, and compare scores according to demographic characteristics.

**Results:**

Of the included 204 participants (mean [SD] age, 26 [7] years; 62 participants from South America [30%]; 32 participants from Mexico [16%]), 117 participants were pregnant (57%), and 87 were within 1-year post partum (43%) at the time of survey completion. Four items were removed based on exploratory factor analysis. Using results from IRT analysis on the remaining 12 items, 5 items were selected to represent communication, caring, competency, accompaniment, and overall trust for the final measure. The 5-item Confianza scale had high measurement precision, with reliability above 0.90 across a wide range of the trust continuum. The Confianza scale (mean [SD] score, 21.5 [4.6] out of 25) was positively correlated with the TPS (*r* = 0.47; 95% CI, 0.36 to 0.57; *P* < .001) and negatively correlated with the EPDS (*r* = −0.41; 95% CI, −0.52 to −0.29; *P* <.001). Higher trust scores were obtained when there was language concordance with clinicians (mean [SD], 23.6 [2.3] vs 20.0 [5.3]; *P* < .001) and care continuity (mean [SD], 22.3 [3.8] vs 20.9 [5.3]; *P* = .001).

**Conclusions and Relevance:**

In this cross-sectional study of pregnant and postpartum Spanish-speaking individuals, a Spanish-language PROM for trust in pregnancy care clinician had initial validity.

## Key Points

**Question:**

Could a psychometrically robust patient-reported outcome measure for patient trust in clinician be developed in Spanish with pregnant and postpartum Spanish-speaking patients?

**Findings:**

In this cross-sectional study of 204 participants, a 5-item scale demonstrated content validity, internal reliability, convergent and discriminant validity, and meaningful associations with mental and physical health outcomes.

**Meaning:**

These findings suggest that a novel patient-reported measure for trust in pregnancy care clinician has initial validity in Spanish.

## Teaser

This cross-sectional study assesses the validity of a Spanish-language patient reported outcome measure (PROM) for trust in pregnancy care clinicians among Spanish-speaking pregnant and postpartum patients.

## Introduction

Trust is an attribute of patient-clinician relationship quality, the vehicle through which evidence-based medicine is delivered, and a key outcome of patient experience.^1^ Trust at all levels of health care is critical to achieving the quintuple aim—better health outcomes, better patient experiences, health equity, reduced costs, and clinician well-being^2^—and has been a spotlight issue magnified by the COVID-19 pandemic.^3,4^ Trust is associated with higher quality of life and satisfaction with care.^5^ Despite the centrality of trust in patient-clinician relationships, 50 years of trust research has revealed conceptual ambiguity in defining and measuring trust.^6^ While the literature on trust across individuals and organizations is nascent, there is a dearth of evidence about building trust between patients and clinicians across language differences. Patients with limited English proficiency experience less access to care,^7^ more adverse events,^8^ and worse quality of care^9^ than English-proficient patients, which may threaten trust-building. Pregnancy is the ideal window to understand how to measure patient trust in clinicians through multiple outpatient visits that usually culminate in a life-changing hospitalization.

In the United States, racial inequities in pregnancy-associated morbidity and mortality are seen in negative experiences of pregnancy care among individuals from minoritized racial and ethnic groups who are at higher risk of mistreatment and discrimination.^10,11,12^ Despite the growing recognition that patient experiences of respectful care—a fundamental foundation for building trust—is needed,^13^ there are few validated patient-reported experience measures (PREMs) or patient-reported outcome measures (PROMs) used in pregnancy.^14^ The Agency for Healthcare Research and Quality published a comparative effectiveness review that found there was no single best measure for respectful maternity care due to overlapping themes and definitions, differing care contexts, and limited evaluation.^15^

While there are multiple measures for patient trust in clinician,^16^ none have been developed in languages other than English or consider the unique context of pregnancy. Of the few validated measures for patient experience in pregnancy care, the vast majority are developed and validated in English, and only a small subset are rigorously translated, culturally adapted, and validated in other languages.^15,17^ Language is important to fully capture the breadth and depth of patient experiences. However, developing rigorous PREMs and PROMs in other languages has been underexplored due to the hegemony of English and lack of research infrastructure to support multilingual approaches to patient-centered outcomes research.^18^ The purpose of this study was to develop a patient-reported measure for patient trust in pregnancy care clinician in Spanish and provide evidence of its initial validity.

## Methods

This cross-sectional study was approved by the Committee on Clinical Investigations at Beth Israel Deaconess Medical Center. The Strengthening the Reporting of Observational Studies in Epidemiology (STROBE) reporting guideline was followed, and informed consent was obtained at the beginning of the survey.

This is a multimethod study to create a psychometrically robust patient-reported outcome measure for trust in pregnancy care clinician in Spanish. We developed the instrument using the Patient-Reported Outcomes Measurement Information System (PROMIS) methodology.^19^ We did an extensive literature review of existing scales to measure trust, including a recently published synthetic review of trust research and a compendium of trust measures.^6,16^ None of the existing instruments were specific to pregnancy or to populations that face language barriers. Therefore, we developed a novel item set for the *Confianza* (Trust) scale based on a qualitative study that explored patient-clinician trust in pregnancy care with Hispanic or Latine patients in Spanish.^20^ After completing focus group discussions, 3 researchers independently coded transcripts in both English and Spanish.^20^ The dimensions identified by the focus groups were communication, caring, competency, comfort, and accompaniment.^20^ Three items were developed for each of the 5 trust constructs based on findings from focus group transcripts in Spanish.^20^ We kept the same minimum number of candidate items for each construct to ensure equal representation of the 5 constructs. All response options were based on a 5-point Likert scale for the frequency that the statement is true, in which 1 was never and 5 was always. We selected a 5-point response scale based on the rationale provided in the original testing of the PROMIS.^21^ We included one overall trust item: “On a scale of 1 to 10, how much do you trust your doctor/midwife?” These responses were recoded to be on a 1 to 5 scale by dividing by 2 and rounding up (eg, 1 or 2 = 1; 3 or 4 = 2). Using the Fernandez Huerta readability index,^22^ we revised the text such that the final items were written at the eighth grade level or below. Subsequently, the research team held 2 meetings with a community advisory board, including Spanish-speaking patients and cultural brokers, to establish face validity and solicit feedback on the candidate items for the Confianza scale. We led 8 cognitive debriefing interviews with pregnant Spanish-speaking patients to evaluate comprehension, recall and retrieval, judgment, and response.^23^ Each interview, ranging from 10 to 20 minutes in duration, involved participants selected based on the same eligibility criteria established for this study. We refined the items and response options in the candidate set accordingly.

### Online Panel Participants

Participants were recruited through CloudResearch,^24^ an online participant recruitment company that uses market research platforms to engage participants who meet specific criteria. Eligibility criteria included being aged 18 years or older, self-identifying as Hispanic or Latine, being currently pregnant or having given birth in the previous 12 months, reporting Spanish as preferred language and limited English proficiency (defined as speaking English less than very well), and living in the US. After potential participants accepted the study invitation, they completed screening questions to verify their eligibility. Those who met the inclusion criteria could continue with the remainder of the survey. Participants received up to $24 for survey completion according to agreed-upon policies from the online participant recruitment company.

### Data Collection

The survey was launched using Prime Panels, a market-research recruitment platform,^25^ from January to May 2024. We chose this as the survey platform because of the diversity of its online panels.^26^ Participants accessed the survey via the online participant recruitment company platform and then were redirected to Research Electronic Data Capture, a Health Insurance Portability and Accountability Act-compliant secure platform for data collection and analysis,^27^ to answer the survey. The survey consisted of 75 questions in 6 sections. At the beginning of the survey, demographic data, language proficiency, and the Brief Acculturation Scale for Hispanics^28^ were collected.

Confianza scale candidate items and the following validated instruments were administered in Spanish (all were developed in English and have published Spanish translations). The Trust in Physician Scale (TPS), developed by Anderson and Dedrick in 1990,^29^ is the most widely used measure for patient trust in clinicians.^6^ The TPS is an 11-item instrument in which responses are measured on a 5-point scale from strongly disagree to strongly agree. Internal reliability is excellent,^29^ and test-retest reliability has been established.^30^ The Edinburgh Postpartum Depression Scale (EPDS), developed by Cox et al^31^ in 1987, is a self-administered questionnaire consisting of 10 items on a 4-point scale to screen for postpartum depression. This scale is widely used during pregnancy through 6 to 8 weeks after birth. The Mothers on Respect (MOR) index is a 14-item index that measures respectful care. The MOR index is a patient-informed quality and safety indicator that can assess clinician-patient relationships and access to person-centered care.^32^ Patients respond based on their interactions with their pregnancy care clinicians. The PROMIS Global-10^33^ is a 10-item scale used to assess global physical and mental health-related quality of life.^21^

The TPS was selected as a comparator measure for convergent validity because it is the most widely used trust scale.^34^ The EPDS and MOR were selected as comparator measures for convergent and discriminant validity because they were specifically developed to measure related constructs of depression and respect, respectively, in pregnant and postpartum populations. The PROMIS Global-10 was selected as a comparator because it is widely recognized as a generalizable measure for physical and mental health across populations.

### Statistical Analysis

Based on published data quality metrics,^26^ we assessed survey completion times and excluded any participants who completed the survey in less than 2 seconds per question (2.43 minutes). We first sought to evaluate and reduce the item set based on examining the underlying dimensionality through Exploratory Factor Analysis (EFA) using Mplus.^35^ We examined eigenvalues to determine the number of factors. We used standard criteria to evaluate the model, including the Comparative Fit Index (CFI) and Tucker-Lewis Index (TLI) with values greater than 0.95 indicating very good model fit. Items that loaded poorly on the single factor in the EFA were removed. We then calibrated the remaining items using item response theory (IRT) to identify the best possible subset of items to represent the construct of trust in pregnancy care clinician.^36^ IRT is a collection of psychometric modeling techniques that characterize items and total scores on the same latent measurement scale. The model-estimated item parameters include a slope (*a* parameter), indicating how associated an item is with the latent construct being measured, and 1 or more location (*b*) parameters that indicate the point on the score continuum that the item responses provide the most information. The slope is analogous to an item-total correlation for which higher is better. Higher location values reflect the need for a higher degree of the measured construct for endorsement. The fact that the items and the individual scores are placed on the same latent scale makes the IRT approach quite versatile in evaluating item and scale properties. IRT also lends itself well to graphical representations of item and scale measurement precision (ie, information curves) which can be examined to select optimal subsets of items for specific measurement purposes. In this study, we used the graded response model^36,37,38^ for items with 3 or more response options and the IRTPRO software^39^ to investigate each candidate item’s parameters and their functioning together as a scale (eg, test information and precision at different levels of the latent trait). We considered item-level fit based on the S-X^2^ index^40,41^ using a *P* < .01 significance level to indicate potential misfit and examined the standardized local dependence χ^2^ statistics^42^ to detect violations of local dependence. We selected the final item set based on content from formative qualitative work with the population of focus^20^ and psychometric performance. We aimed to limit the final scale to 5 items to ensure feasible application in routine clinical care and scalability with the highest possible response rate. We examined information curves to compare psychometric performance of item sets within each trust domain. Information curves are an output of an IRT calibration that display the amount of measurement information an item or set of items provides (y-axis) across the underlying score continuum (x-axis). Information values reflect measurement precision such that information values of 10, 5, and 3.5 correspond to reliability values of 0.90, 0.80, and 0.71, respectively. This association allows for clear interpretation of the precision tradeoffs associated with selection of 1 set of items over another.

Once the final scale was established, we examined reliability, content validity, and construct validity (convergent, discriminant, and known-groups validity).^43^ We did not measure criterion validity because there is no consensus about which outcome measure is most appropriate for this type of validity regarding trust in pregnancy care clinician. We measured item-total correlations and internal consistency with Cronbach α, item and total scale mean scores, and floor and ceiling effects. We also evaluated convergent and discriminant validity using Pearson correlations of the Confianza scale with TPS, EPDS, MOR, PROMIS Global-10 physical and mental health summary scores. We hypothesized that the Confianza scale would be negatively associated with the EPDS and positively associated with the TPS, MOR, and PROMIS Global-10 scores. We also performed known-groups validity and hypothesized that patients who had frequent language concordance (defined as the frequency of speaking their preferred language with their clinician often or always) and clinician continuity (defined as seeing the same clinician often or always) would have higher mean Confianza scores than patients with lower language concordance and clinician continuity based on previous formative work.^20,44,45,46^ Data were collected from January to May 2024 using STATA version 17 (StataCorp). Statistical significance was set at *P* < .05, and tests were 2-sided.

## Results

### Sample Description 

There were 855 respondents, of which 272 were eligible to participate (Figure). Among those eligible, 68 were excluded, resulting in 204 participants (Figure). The 62 excluded participants who did not complete the survey did not differ from the included sample (eTable 1 in Supplement 1). The final sample of respondents had a mean (SD) age of 26 (7) years, 62 participants reported being from South America (30%), 32 reported being from Mexico (16%), and 17 reported being from the United States (8%) (Table 1). Additionally, 128 participants reported having a high school education or less (63%), 117 were pregnant (57%), and 87 had given birth within the last 12 months (43%).

**Figure.**
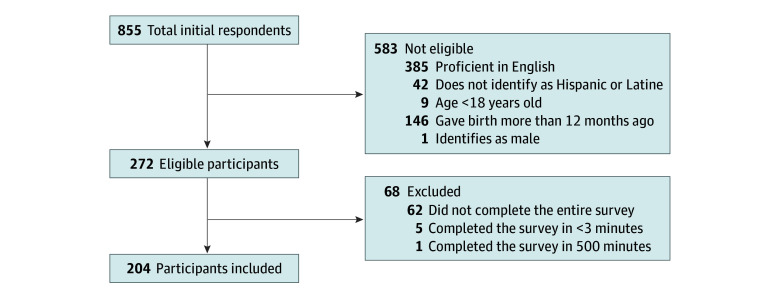
Recruitment Flow Diagram

**Table 1.** Participant Characteristics

Characteristics	Participants, No. (%) (N = 204)
Age, mean (SD)	26 (7)
Region of origin	
South America	62 (30)
Central America	47 (23)
Caribbean	46 (23)
Mexico	32 (16)
United States	17 (8)
Self-reported oral proficiency in English	
Fair, I can hold basic conversations in English, but often use a mix of Spanish and English	70 (34)
Limited, I am limited to a few words or phrases	97 (48)
Not at all, I do not speak any English	37 (18)
Language spoken at home	
Spanish only	180 (88)
Spanish and English	22 (11)
English only	2 (1)
Education	
Less than high school	28 (14)
High school diploma or equivalent	100 (49)
Some college or Bachelor’s degree	59 (29)
Master’s degree	5 (2)
Doctorate or professional degree	12 (6)
Pregnancy status	
Pregnant	117 (57)
Weeks of gestation, mean (SD)	20 (12)
Post partum	87 (43)
<1, mo	48 (55)
1-12, mo	39 (45)
Complications^a^	
None	129 (63)
Anxiety	31 (15)
Depression	26 (13)
High blood pressure or preeclampsia	12 (6)
Diabetes	7 (3)
Preterm birth	4 (2)
Spontaneous abortion	4 (2)
Other	4 (2)
No. of visits	
0	38 (19)
1	25 (12)
2-3	59 (29)
4-5	34 (17)
>5	48 (24)
Clinician continuity	
Yes, I always or almost always saw the same doctor or midwife	127 (62)
Sometimes, I sometimes saw the same doctor or midwife	54 (27)
No, I never or almost never saw the same doctor or midwife	23 (11)
Preferred language to communicate with clinician	
Spanish	191 (94)
English	13 (6)
Frequency of utilizing the preferred language with clinician	
Never	31 (15)
Rarely	43 (21)
Sometimes	42 (21)
Often	40 (20)
Always	48 (24)
Language used to communicate with clinician	
We both spoke English	11 (6)
We both spoke Spanish	113 (55)
We spoke both English and Spanish with an interpreter	67 (33)
We spoke both English and Spanish without an interpreter	13 (6)

^a^ More than 1 response possible.

### Item Reduction and Final Scale Selection 

Four items (question 3, question 5, question 6, and question 13) were removed from the item set based on low factor loadings from an initial EFA with all 16 items (Table 2). The EFA with the remaining 12 items yielded a single eigenvalue greater than 1, and a reasonable fit (CFI, 0.97; TLI, 0.97), confirming the essential unidimensionality of the 12-item set. The graded response model IRT calibration of the 12 items revealed 1 item with poor fit (question 14: S-X^2^ = 44.42; *df* = 25; *P* = .0097). All other items exhibited acceptable fit, did not display local dependence, and had reasonable item parameters with slopes ranging from 2.52 to 5.39 and average location parameters ranging from −1.36 to −0.87 (eTable 2 in Supplement 1). We made a series of decisions to reduce the remaining 11 items down to 5 for the final scale. First, we decided to include the overall trust item (question 16) due to its strong content validity. We also opted to exclude the single remaining item from the comfort domain (question 4) based on both empirical and content considerations. Specifically, question 4 had a relatively low slope, and the comfort domain was considered less important conceptually in patient focus groups. To decide on the remaining 4 items, we examined item information curves to compare the measurement precision of the of the items, grouping them according to their domains (eFigure 1 in Supplement 1) and selected the item within each domain with the strongest psychometric properties (question 2: communication, ie, being honest; question 7: caring, ie, feels safe and valued; question 11: accompaniment, ie, looks out for me; and question 15: competency, ie, trust recommendations). Results of a final IRT calibration of the 5-item Confianza scale indicate that the scale displayed strong psychometric properties (eTable 3 in Supplement 1), with a reliability of 0.90 or higher for a large range of the trust continuum (from approximately 2.2 SD below the mean to 0.2 SD above the mean) (eFigure 2 in Supplement 1).

**Table 2.** Abbreviated Content of *Confianza* Scale Initial Item Set and Factor Loadings

Question	Spanish^a^	English^b^	Dimension of trust	EFA factor loadings (SE) 16 items	EFA factor loadings (SE) 12 items
1	Brindar información clara	Gives clear information	Communication	0.82 (0.03)	0.82 (0.03)
2	Ser honesto	Being honest	Communication	0.89 (0.02)^c^	0.89 (0.02)
3	No juzgar	Does not judge	Communication	0.15 (0.07)^d^	NA
4	Compartir dudas	Shares concerns	Comfort	0.82 (0.03)	0.81 (0.03)
5	Ser paciente	Being patient	Comfort	0.61 (0.04)^d^	NA
6	Mantener confidencialidad	Maintains confidentiality	Comfort	0.61 (0.05)^d^	NA
7	Sentirse segura, escuchada, plena y valorada	Feels safe, heard, whole, and valued	Caring	0.94 (0.01)^c^	0.94 (0.01)
8	Preocuparse por mí	Cares about me	Caring	0.92 (0.02)	0.92 (0.02)
9	Respetarme	Respects me	Caring	0.89 (0.02)	0.89 (0.02)
10	Poder contar con mi doctor(a)/partera	Being dependable	Accompaniment	0.81 (0.03)	0.81 (0.03)
11	Estar pendiente de mí	Looks out for me	Accompaniment	0.87 (0.02)^c^	0.88 (0.02)
12	Estar al lado mío	Being by my side	Accompaniment	0.82 (0.03)	0.82 (0.03)
13	Es experto en el embarazo	Being an expert in pregnancy care	Competency	0.15 (0.05)^d^	NA
14	Ofrecer atención de buena calidad	Provides good quality care	Competency	0.88 (0.02)	0.88 (0.02)
15	Confiar en las recomendaciones	Trust recommendations	Competency	0.89 (0.02)^c^	0.89 (0.02)
16	Confianza total (1, no confiar, 10, confianza absoluta)^e^	Overall trust (1, do not trust; 10, absolute trust)	Overall trust	0.84 (0.04)^c^	0.84 (0.04)

Abbreviations: EFA, exploratory factor analysis; NA, not applicable.

^a^ Spanish response options for Q1-15: (1) nunca; (2) casi nunca; (3) a veces; (4) a menudo; (5) siempre.

^b^ English response options for Q1-15: (1) never; (2) rarely; (3) sometimes; (4) often; (5) always.

^c^ Selected for the final scale.

^d^ Removed due to low factor loadings.

^e^ These responses were recoded to be on a 1 to 5 scale by dividing by 2 and rounding up (eg, 1 or 2 = 1; 3 or 4 = 2).

### Scale Evaluation 

The 5-item set demonstrated good internal reliability with item-total correlations ranging from 0.70 to 0.86 and Cronbach α of 0.91. The overall mean (SD) score of the 5-item scale was 21.5 of 25 (4.6) (Table 3 and eFigure 3 in Supplement 1). The floor effect was negligible (3% [6 of 204]); however, there was a substantial ceiling effect of 48% (76 of 204). Despite the ceiling effect, the scale varied meaningfully according to demographic and clinical characteristics (Table 3). While there were no statistically significant differences in trust scores by education level, there were higher trust scores among participants who were pregnant vs post partum (mean [SD] score, 22.1 [3.8] vs 20.7 [5.4]; *P* = .03) and who reported language concordance with their clinician (mean [SD] score, 23.6 [2.3] vs 20.0 [5.3]; *P* < .001) and continuity with the same clinician (mean [SD] score, 22.3 [3.8] vs 20.9 [5.3]; *P* < .001). Scores also differed significantly according to screening results on the EPDS for negative and positive screens (mean [SD] score, 23.0 [23.8] vs 20.1 [19.2]; *P* = .001), high vs low scores on the PROMIS Global-10 mental and physical health-related quality of life summary scores (mental: mean [SD] score, 22.1 [4.5] vs 20.6 [4.7]; *P* = .03; physical: mean [SD] score, 22.5 [4.4] vs 19.7 [4.6]; *P* < .001). Finally, the 5-item Confianza scale was moderately correlated with the TPS (*r* = 0.47; 95% CI, 0.36 to 0.57; *P* < .001), MOR index (*r* = 0.33; 95% CI, 0.20 to 0.44; *P* < .001), and PROMIS-Global 10 scales (mental health: *r* = 0.26; 95% CI, 0.12 to 0.38; *P* < .001; physical health: *r* = 0.25; 95% CI, 0.11 to 0.38; *P* < .001) and negatively correlated with the EPDS (*r* = −0.41; 95% CI, −0.52 to −0.29; *P* < .001) (Table 4).

**Table 3.** *Confianza* Scores by Participant Demographics and Clinical Characteristics

Participant characteristic	Participants, No. (%)	Score, mean (SD)	*P* value
Overall *Confianza* score	204	21.5 (4.6)	NA
Item 2—being honest	NA	4.2 (1.1)	
Item 7—feels safe, heard, whole, and valued	NA	4.3 (1.1)	
Item 11—looks out for me	NA	4.2 (1.0)	
Item 15—trust recommendations	NA	4.3 (1.1)	
Item 16—overall trust (1, do not trust; 10, absolute trust)^a^	NA	4.5 (1.0)	
Pregnancy status			
Pregnant	117 (57)	22.1 (3.8)	.03
Post partum	87 (43)	20.7 (5.4)	
Education			
Low education^b^	128 (63)	21.7 (4.4)	.45
High education^c^	76 (37)	21.2 (4.9)	
Language concordance with clinician			
Often or always	88 (43)	23.6 (2.3)	<.001
Sometimes, rarely or never	116 (57)	20.0 (5.3)	
Continuity with same clinician			
Often or always	127 (62)	22.3 (3.8)	<.001
Sometimes, rarely or never	77 (38)	20.9 (5.3)	
Edinburgh Postpartum Depression Scale			
Score ≥10 (screen positive)	104 (51)	20.1 (19.2)	<.001
Score <10 (screen negative)	100 (49)	23.0 (23.8)	
PROMIS-Global 10			
Low mental health	75 (37)	20.6 (4.7)	.03
High mental health	129 (63)	22.1 (4.5)	
PROMIS-Global 10			
Low physical health	68	19.7 (4.6)	<.001
High physical health	117	22.5 (4.4)	

Abbreviations: NA, not applicable; PROMIS, Patient-Reported Outcomes Measurement Information System.

^a^ These responses were recoded to be on a 1 to 5 scale by dividing by 2 and rounding up (eg, 1 or 2 = 1; 3 or 4 = 2, etc).

^b^ Low education includes less than high school and high school diploma or equivalent.

^c^ High education includes some college or Bachelor’s degree, masters degree, or doctorate or professional degree.

**Table 4.** Convergent and Discriminant Validity of the 5-Item *Confianza* Scale

Scale	Pearson correlation coefficients (95% CI)	*P* value
Trust in Physician Scale	0.47 (0.36 to 0.57)	<.001
Mothers on Respect Index	0.33 (0.20 to 0.44)	<.001
Edinburgh Postpartum Depression Scale	−0.41 (−0.52 to −0.29)	<.001
PROMIS-Global Mental Health	0.26 (0.12 to 0.38)	<.001
PROMIS-Global Physical Health	0.25 (0.11 to 0.38)	<.001

Abbreviation: PROMIS, Patient-Reported Outcomes Measurement Information System.

## Discussion

These findings suggest that the novel Confianza scale was a psychometrically robust Spanish PROM for trust in clinician during pregnancy care. The development process of the Confianza scale was novel in its empirical development in Spanish. We found meaningful differences in trust scores based on patient-clinician dyad factors (language concordance) and health system factors (clinician continuity). We also found meaningful differences in trust scores based on physical and mental health outcomes, which was consistent with other studies.^5^

This study presented an equity-driven approach to understanding and measuring patient experiences in a linguistically minoritized population that faces inequities in pregnancy care due to language barriers and policy environments.^47^ There was a paucity of PREMs and PROMs in pregnancy care and no national consensus on which instruments should be applied across settings. While multiple population-based survey studies^10,48^ have examined pregnancy and childbirth experiences, these were often difficult to operationalize in learning health systems and clinical settings due to the length of the surveys.^49^ We aimed for simplicity given the myriad challenges with implementing PREMs and PROMs,^50^ especially in busy obstetric care practices.^51^ Based on data from a diverse online panel of participants, we developed a 5-item scale that favors simplicity and feasibility over comprehensiveness of measuring all concepts and dimensions of trust. We demonstrated high internal reliability for the items and convergent and discriminant validity through the positive correlation with the widely used TPS and the negative correlation with the EPDS.

While a purpose of PROMs was to better understand how patients were feeling and experiencing their health and health care, another important purpose was to guide clinical communication during patient encounters and quality improvement.^52^ Three of the 5 items pointed to specific clinician behaviors: being honest with patients; looking out for and following up with patients; and making patients feel safe, heard, whole, and valued. These trust-building behaviors among clinicians reinforced the importance of respectful and empathic communication skills, especially when language discordance exists between patients and clinicians. Interpersonal dynamics and relationship-building behaviors were modifiable and created an opportunity to design interventions and tools to enhance patient trust in their clinician even when language barriers were present.

### Limitations

This study has limitations. We used an online panel for participant recruitment, which precluded ascertaining clinical information from medical record systems. Additionally, participants in online panels may be more engaged in research than the average clinical population as a result of differing experiences in the health care system. Further validation is needed in a clinical cohort to confirm sample characteristics and outcomes and establish test-retest reliability and responsiveness. We limited our study population to participants who were either pregnant or had recently given birth at the time of data collection, which may limit generalizability to other populations. We observed a notable ceiling effect of the Confianza scale, consistent with other measures of patients’ evaluations of their clinicians.^53,54^ Despite these limitations, we identified important findings in a sample of patients with Spanish preferred language and limited English proficiency. Our innovative, equity-centered approach challenges the English-dominant paradigm of patient-reported outcomes research by grappling with how language shapes experiences and developing a PROM from empirical data in Spanish. Existing PROMs are limited due to their development from English-speaking populations where important constructs may differ from other linguistic populations. Additionally, not all PROMs are translated into other languages and there is inconsistent quality with variability in translation, back translation, and cultural adaptation.

## Conclusions

This cross-sectional study presented the initial validity of a novel 5-item Confianza scale, which is among the first PROMs for trust in clinician to be developed and validated in Spanish. This study is an illustrative example of applying language justice for the largest linguistic minority in patient-centered outcomes research. Future work should include further validation in a clinical cohort of Spanish-speaking patients, validation of the English translation, and applying multilingual research methods to other PROMs.

## Notes

Supplement 1. **eTable 1.** Participant Demographics (Excluded Due to Incomplete Responses vs Included) **eTable 2.** IRT Parameters for 12 Candidate Confianza Scale Items **eTable 3.** IRT Parameters for Final 5-item Confianza Scale **eFigure 1.** Item Information Curves for Communication, Caring, Confianza Candidate Items **eFigure 2.** Information Curve for Final 5-Item Set (Items 2, 7, 11, 15, and 16) **eFigure 3. **Confianza Scale Item Response Frequencies

Supplement 2.
Data Sharing Statement
