# Supplementary material for: A Spanish-Language Patient-Reported Outcome Measure for Trust in Pregnancy Care Clinician
Source: JAMA Netw Open. 2025 Feb 18;8(2):e2460465. doi: 10.1001/jamanetworkopen.2024.60465 (PMC11836756; doi:10.1001/jamanetworkopen.2024.60465)
Supplement: Supplement 1. — eTable 1. Participant Demographics (Excluded Due to Incomplete Responses vs Included) eTable 2. IRT Parameters for 12 Candidate Confianza Scale Items eTable 3. IRT Parameters for Final 5-item Confianza Scale eFigure 1. Item Information Curves for Communication, Caring, Confianza Candidate Items eFigure 2. Information Curve for Final 5-Item Set (Items 2, 7, 11, 15, and 16) eFigure 3. Confianza Scale Item Response Frequencies [file jamanetwopen-e2460465-s001.pdf]

Supplemental Online Content

Molina RL, Bazan M, Hacker MR, et al. Development of a patient-reported outcome measure for trust in pregnancy care clinician with spanish-speaking patients. *JAMA Netw Open*. 2025;8(2):e2460465. doi:10.1001/jamanetworkopen.2024.60465

eTable 1. Participant Demographics (Excluded Due to Incomplete Responses vs Included)
eTable 2. IRT Parameters for 12 Candidate *Confianza* Scale Items
eTable 3. IRT Parameters for Final 5-item *Confianza* Scale
eFigure 1. Item Information Curves for Communication, Caring, Confianza Candidate Items
eFigure 2. Information Curve for Final 5-Item Set (Items 2,7,11,15, and 16)
eFigure 3. *Confianza* Scale Item Response Frequencies

This supplemental material has been provided by the authors to give readers additional information about their work.

© 2025 Molina RL et al. *JAMA Network Open*.

eTable 1. Participant Demographics (excluded due to incomplete responses vs included)

Characteristics	Excluded (N=67)	Included (N=204)
Age, mean (SD)	24 (6)	26 (7)
Pregnancy status		
Pregnant	41 (62)	117 (57)
Weeks of gestation, mean (SD)**	19 (38)	20 (12)
Postpartum***		
<1 month	9 (45)	48 (55)
1-12 months	11 (55)	39 (45)
Education		
Less than high school	15 (23)	28 (14)
High school diploma or equivalent	29 (43)	100 (49)
Some college/ Bachelor's degree	17 (25)	59 (29)
Master's degree	1 (1)	5 (2)
Doctorate or professional degree	5 (7)	12 (6)
Clinician continuity ***		
Yes – I always or almost always saw the same doctor or midwife	33 (53)	127 (62)
Sometimes – I sometimes saw the same doctor or midwife	15 (24)	54 (27)
No – I never or almost never saw the same doctor or midwife	14 (11)	23 (11)
Frequency of utilizing the preferred language with clinician***		
Never	12 (20)	31 (15)
Rarely	11 (18)	43 (21)
Sometimes	12 (20)	42 (21)
Often	11 (18)	40 (20)
Always	15 (25)	48 (24)

*1 missing response missing from the excluded group.

**2 missing responses from the excluded group.

***5 or 6 missing responses from the excluded group.

© 2025 Molina RL et al. *JAMA Network Open*.

eTable 2. IRT Parameters for 12 Candidate *Confianza* Scale Items

English Item Content	*a (se)*	b_1 *(se)*	b_2 *(se)*	b_3 *(se)*	b_4 *(se)*
Gives clear information	2.68 (0.35)	-1.81 (0.21)	-1.18 (0.14)	-0.60 (0.12)	0.10 (0.12)
Being honest*	3.31 (0.45)	-1.95 (0.22)	-1.50 (0.16)	-0.82 (0.12)	-0.22 (0.11)
Shares concerns	2.74 (0.37)	-1.92 (0.22)	-1.51 (0.17)	-0.91 (0.13)	-0.13 (0.12)
Feels safe, heard, whole, and valued*	5.39 (0.83)	-1.51 (0.16)	-1.29 (0.13)	-0.92 (0.11)	-0.21 (0.11)
Cares about me	4.45 (0.63)	-1.94 (0.23)	-1.47 (0.15)	-0.86 (0.11)	-0.19 (0.11)
Respects me	4.00 (0.58)	-1.84 (0.21)	-1.66 (0.18)	-1.02 (0.12)	-0.39 (0.11)
Being dependable	2.52 (0.33)	-2.04 (0.24)	-1.44 (0.17)	-0.94 (0.13)	-0.15 (0.12)
Looks out for me*	3.52 (0.46)	-1.91 (0.22)	-1.56 (0.17)	-0.89 (0.12)	-0.06 (0.11)
Being by my side	2.74 (0.35)	-1.89 (0.22)	-1.45 (0.17)	-0.75 (0.12)	0.08 (0.12)
Provides good quality care	3.07 (0.41)	-1.99 (0.23)	-1.46 (0.17)	-1.07 (0.13)	-0.16 (0.11)
Trust recommendations*	3.54 (0.48)	-2.02 (0.23)	-1.30 (0.14)	-0.91 (0.12)	-0.28 (0.11)
Overall trust (1: Do not trust, 10: Absolute trust)*	2.89 (0.43)	-1.84 (0.22)	-1.71 (0.20)	-1.29 (0.15)	-0.59 (0.12)

* Indicates the selected 5 items for the *Confianza* scale.

© 2025 Molina RL et al. *JAMA Network Open*.

eTable 3. IRT Parameters for Final 5-item *Confianza* Scale

English Item Content	*a (se)*	b_1 *(se)*	b_2 *(se)*	b_3 *(se)*	b_4 *(se)*
Being honest	3.62 (0.55)	-1.97 (0.20)	-1.55 (0.16)	-0.85 (0.11)	-0.24 (0.10)
Feels safe, heard, whole, and valued	4.56 (0.58)	-1.71 (0.16)	-1.45 (0.14)	-1.01 (0.11)	-0.23 (0.09)
Looks out for me	2.73 (0.38)	-2.08 (0.23)	-1.75 (0.19)	-1.01 (0.13)	-0.05 (0.10)
Trust recommendations	2.94 (0.42)	-2.13 (0.23)	-1.44 (0.16)	-1.01 (0.12)	-0.31 (0.10)
Overall trust (1: Do not trust, 10: Absolute trust)	2.58 (0.40)	-1.98 (0.23)	-1.86 (0.21)	-1.41 (0.16)	-0.65 (0.11)

© 2025 Molina RL et al. *JAMA Network Open*.

eFigure 1. Item Information Curves for Communication, Caring, *Confianza* Candidate Items

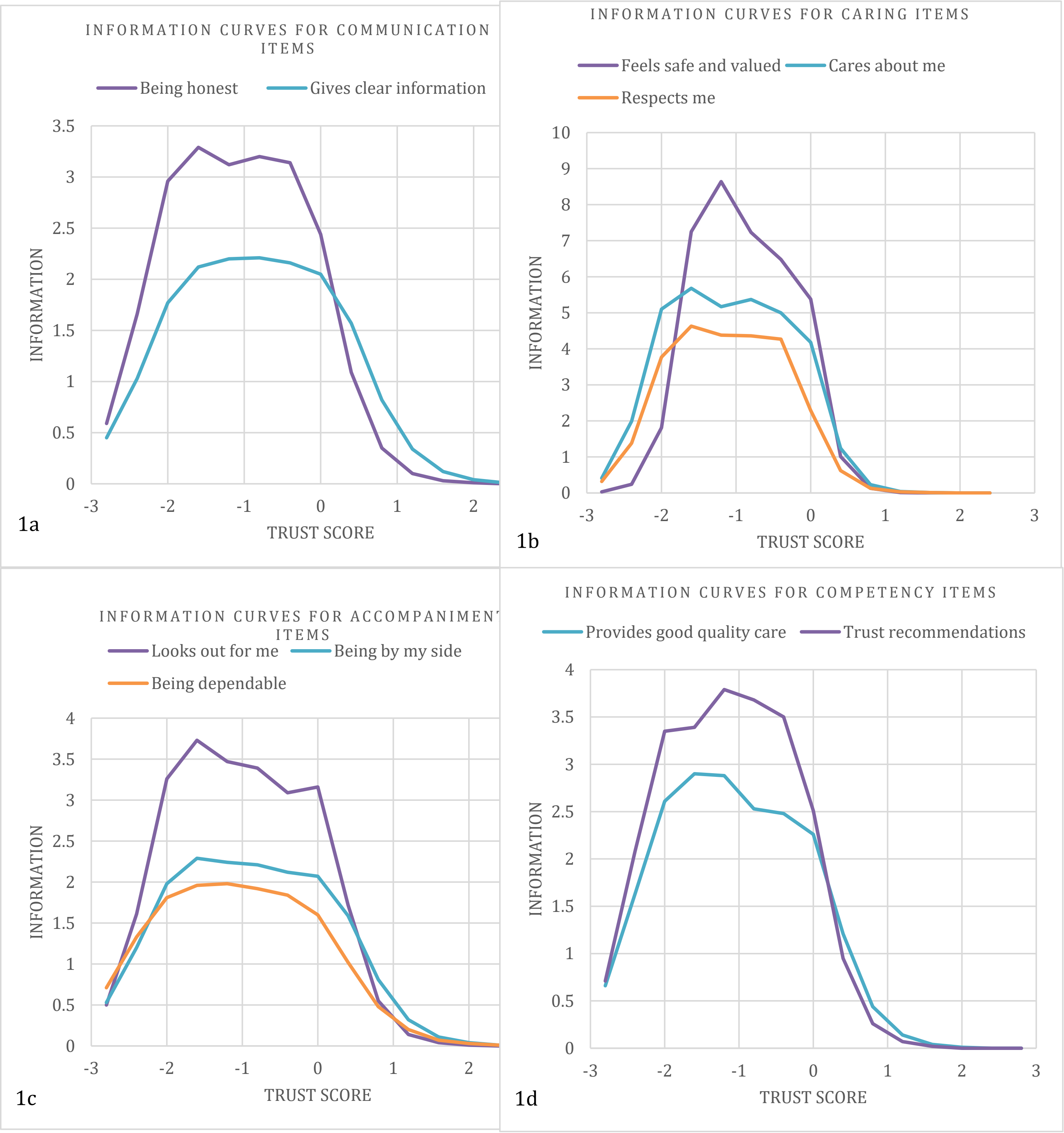

© 2025 Molina RL et al. *JAMA Network Open*.

eFigure 2. Information Curve for Final 5-item Set (2,7,11,15,16)

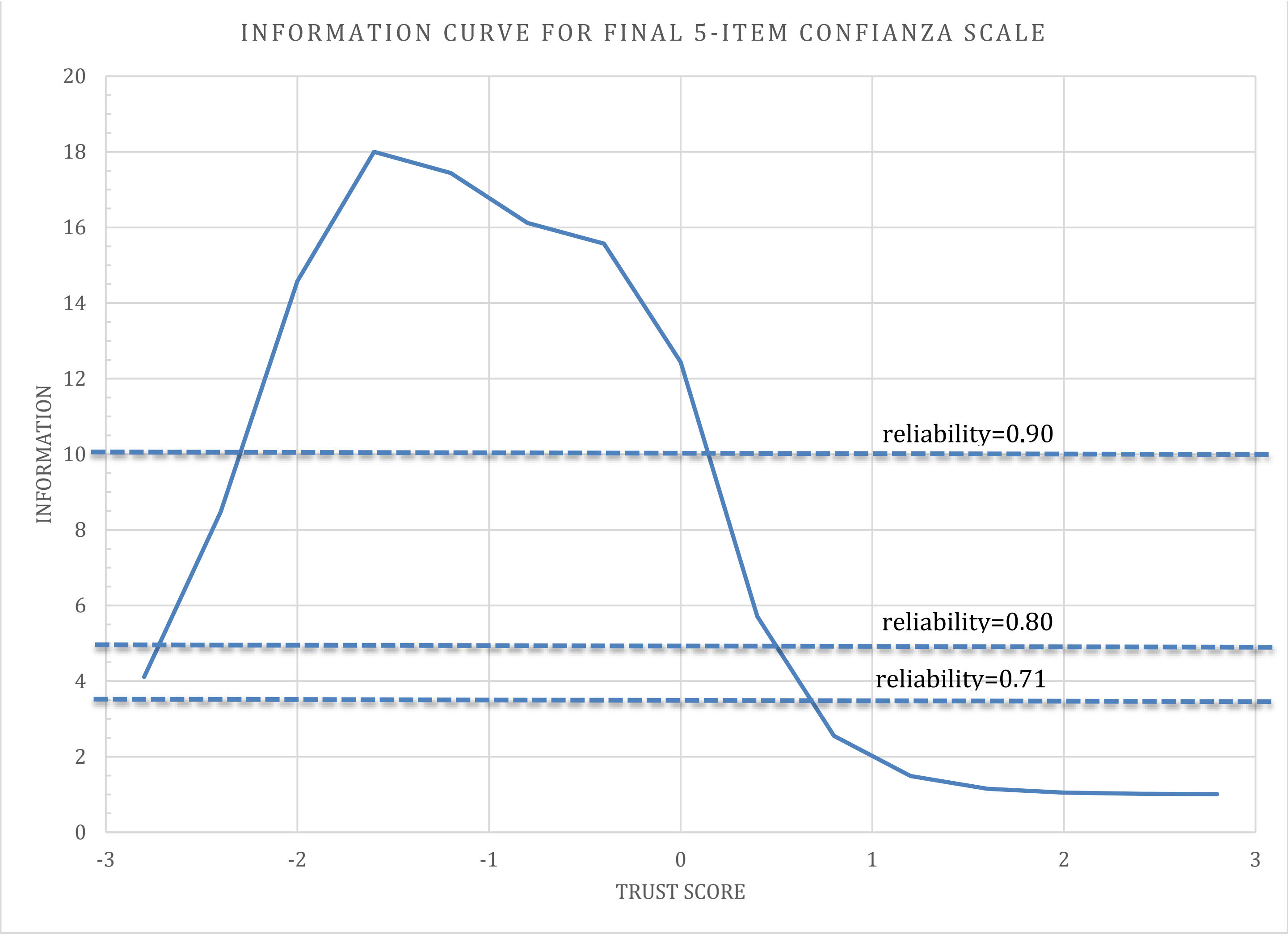

© 2025 Molina RL et al. *JAMA Network Open*.

eFigure 3. *Confianza* Scale Item Response Frequencies

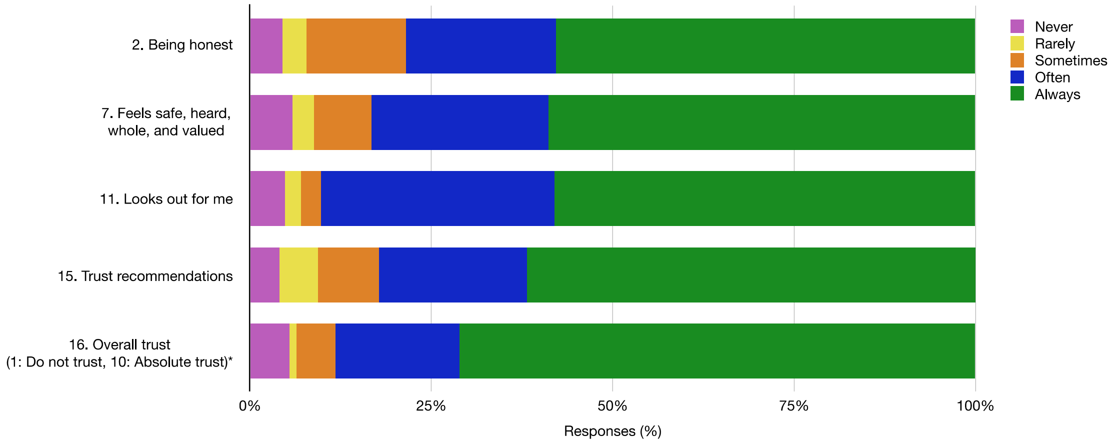

© 2025 Molina RL et al. *JAMA Network Open*.
