# Supplementary material for: A Spanish-Language Patient-Reported Outcome Measure for Trust in Pregnancy Care Clinician
Source: JAMA Netw Open. 2025 Feb 18;8(2):e2460465. doi: 10.1001/jamanetworkopen.2024.60465 (PMC11836756; doi:10.1001/jamanetworkopen.2024.60465)
Supplement: Supplement 2. — Data Sharing Statement [file jamanetwopen-e2460465-s002.pdf]

Data Sharing Statement

Molina. A Spanish-Language Patient-Reported Outcome Measure for Trust in Pregnancy Care Clinician. *JAMA Netw Open*. Published February 18, 2025.
doi:10.1001/jamanetworkopen.2024.60465

Data
Data available: Yes
Data types: Deidentified participant data, Data dictionary
How to access data: A de-identified dataset will be made available upon request and after appropriate data use agreements and human subjects approval are in place. To request access to the data, please email Dr. Rose Molina at rmolina@bidmc.harvard.edu.
When available: With publication

Supporting Documents
Document types: None

Additional Information
Who can access the data: Researchers whose proposed use of the data has been approved.
Types of analyses: For any specified purpose with the appropriate institutional approvals (data use agreement and human subjects approval).
Mechanisms of data availability: With human subjects approval and data use agreement.
